# Optimization of the Transwell^®^ System for Assessing the Dissolution Behavior of Orally Inhaled Drug Products through In Vitro and In Silico Approaches

**DOI:** 10.3390/pharmaceutics13081109

**Published:** 2021-07-21

**Authors:** Elham Amini, Abhinav Kurumaddali, Sharvari Bhagwat, Simon M. Berger, Günther Hochhaus

**Affiliations:** Department of Pharmaceutics, College of Pharmacy, University of Florida, 1245 Center Drive, Gainesville, FL 32610, USA; elham.amini@ufl.edu (E.A.); kuruabhinav@gmail.com (A.K.); sharvari.bhagwat@gmail.com (S.B.); simonberger@ufl.edu (S.M.B.)

**Keywords:** orally inhaled drug products, dissolution, Transwell^®^, sample preparation, anatomical mouth–throat model, mathematical modelling

## Abstract

The aim of this study was to further evaluate and optimize the Transwell^®^ system for assessing the dissolution behavior of orally inhaled drug products (OIDPs), using fluticasone propionate as a model drug. Sample preparation involved the collection of a relevant inhalable dose fraction through an anatomical mouth/throat model, resulting in a more uniform presentation of drug particles during the subsequent dissolution test. The method differed from previously published procedures by (1) using a 0.4 µm polycarbonate (PC) membrane, (2) stirring the receptor compartment, and (3) placing the drug-containing side of the filter paper face downwards, towards the PC membrane. A model developed in silico, paired with the results of in vitro studies, suggested that a dissolution medium providing a solubility of about 5 µg/mL would be a good starting point for the method’s development, resulting in mean transfer times that were about 10 times longer than those of a solution. Furthermore, the model suggested that larger donor/receptor and sampling volumes (3, 3.3 and 2 mL, respectively) will significantly reduce the so-called “mass effect”. The outcomes of this study shed further light on the impact of experimental conditions on the complex interplay of dissolution and diffusion within a volume-limited system, under non-sink conditions.

## 1. Introduction

Differences in dissolution rates have shown to affect the pulmonary residence time of orally inhaled lipophilic drugs such as fluticasone propionate and mometasone furoate [1]. Despite the relevance of the dissolution process for the local and systemic drug exposure of OIDPs, dissolution tests have not yet been incorporated into the arsenal of in vitro tests recommended by regulatory agencies, nor are fully validated methods available [2]. The relevance of dissolution tests for the in vitro assessment of OIDPs was even questioned by a USP Inhalation Ad Hoc Advisory Panel, which “could not find compelling evidence suggesting that such dissolution testing is kinetically and/or clinically crucial for currently approved inhalation products” [2].

However, the pulmonary efficacy of an inhaled drug and the extent of pulmonary targeting is not only affected by deposition-related properties, but also by post-deposition events, such as dissolution and permeation [3]. Realization of the significance of these events for OIDPs led to efforts to classify drugs according to their biopharmaceutical properties [1]. While the need for biorelevant dissolution methods has been recently highlighted [4], stakeholders have not yet agreed on a standardized methodology. Such a method should allow for a study of the dissolution of the drug product’s respirable fraction, to adequately relate the physicochemical characteristics of the drug to its in vivo performance. Published approaches have utilized a range of dose collection and dissolution methodologies [5,6]. Suggested dissolution systems ranged from conventional USP dissolution apparatuses (e.g., USP II or V [7,8], and IV [9,10]), to fluid-limited diffusion-based approaches (e.g., Franz cell [11] and Transwell^®^ [12,13,14]), and Dissolv*It* [15,16], with a range of particle collection methods used for sample preparation [17,18,19,20].

**Figure 1:**
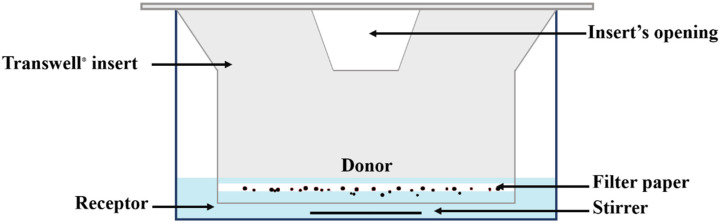
Schematic representation of the Transwell^®^ setup.

Rohrschneider et al. described a method that employed an Anderssson cascade impactor (ACI) for sample preparation, while the Transwell^®^ system was used to monitor the dissolution profile [21]. Within this system, (1) dissolution of the drug particles occurs in a small volume of dissolution medium present in the donor compartment, while (2) the dissolved drug is monitored in the receptor compartment after diffusion across a semi-permeable membrane dividing both compartments (Figure 1). The data generated with this approach have been shown to predict the pulmonary absorption of orally inhaled corticosteroids [22].

In the current study, we further evaluated the Transwell^®^ system as a potentially promising tool for assessing the dissolution characteristics of lipophilic OIDPs. This included testing the suitability of an anatomical mouth/throat model for particle collection, and using in vitro and in silico approaches to assess how experimental conditions (surfactant concentrations, donor/receptor, and sampling volumes) will affect the outcome of the experiments, including the ability to distinguish between samples containing materials of different particle sizes. We were also interested in reducing the so-called “mass effect” observed by others [12,13] when disseminating the developed in silico simulation model through an R Shiny interface, and suggesting more optimized experimental conditions.

## 2. Materials and Methods

### 2.1. Materials

Three previously described investigational capsule-based DPI formulations of fluticasone propionate (Formulation A, B and C; 100 µg/capsule [23]) contained the same batch of fluticasone propionate particles, but differed in lactose content and composition, resulting in different median mass aerodynamic diameters (MMAD). Capsules were delivered via a DPI Monohaler 8^®^ device (Plastiape, Milan, Italy). In addition, Flovent^®^ HFA 44 µg MDI (GlaxoSmithKline plc) and Survanta^®^ were purchased from the University of Florida Investigational Drug Services (IDS) pharmacy. Micronized fluticasone propionate powder was provided by Hovione FarmaCiencia SA (Loures, Portugal). The inhaled formulations are subsequently referred to by their non-trade names (fluticasone propionate (FP)).

Sodium dodecyl sulfate (SDS), Tween^®^ 80, and 24 mm glass microfiber filters (Whatman^®^ GF/C) were purchased from Sigma Aldrich (St. Louis, MO, USA). Six-well 24 mm Transwell^®^ polycarbonate (PC) membrane cell culture inserts with lid (Cat. No. 3412, Corning Inc., Midland, NC, USA) were used for all dissolution and diffusion studies. Acetonitrile (HPLC grade), ethanol (HPLC grade), methanol (HPLC grade), and potassium sulfate were purchased from Fisher Scientific^®^ (Pittsburgh, PA, USA).

A 7-stage Next-Generation Impactor (NGI) from Copley Scientific^®^ (Nottingham, U.K.) was used, equipped with a flow control. A medium-sized VCU (Virginia Commonwealth University) throat model was received from RDD Online LLC (Virginia Biotechnology Research Park, VA, USA). Molykote™ 316 Silicone Release Spray (Midland, MI, USA) was purchased from Dow Corning^®^. Ten-To-One/High Tear Strength Mold Rubber was purchased from Micro-Mark^®^ (Berkeley Heights, NJ, USA) to make mouthpiece adaptors for the VCU throat model.

### 2.2. 3D X-ray Scanning of the Transwell^®^ System

In order to minimize fluid transfer due to hydrostatic pressure differences between the donor and receptor compartment of the Transwell^®^ system, the dissolution medium volumes in the donor and receptor compartment needed to be selected in such a way that both compartments exhibited equal fluid levels. To reach this objective, three-dimensional cross-sectional images of the Transwell^®^ system were obtained through 3D X-ray scanning using a GE v|tome|x m-computed tomography (CT) scanner at ~55 µm voxel size, 90 kV, and 200 mA. Discrete visualization of the scanned object was performed in the native voxel format. Image reconstruction and analysis were performed within the Volume Graphics Studio Max v3.2.4, which specified all surfaces and interior dimensions of the Transwell^®^ system (Table 1). The maximum available volumes for the donor and acceptor compartments were obtained through this step. The volume of the glass microfiber filter used for particle collection was also estimated by the dimensions of the filter reported by the manufacturer. Based on these measurements, matching receptor acceptor and donor volumes providing equal fluid levels were derived and linked via a linear relationship.

### 2.3. Solubility Determinations

Excess amounts of micronized fluticasone propionate powder were weighted into volumetric flasks (10 mg per flask). Flasks were filled with 10 mL of different dissolution media (i.e., Tween^®^ 80 and SDS in distilled water ranging from 0.0 to 5.0% *w*/*v*, and 0.2% *w*/*v* diluted Survanta^®^; see Table 2). The suspended particles were stirred for 24 h at 400 rpm and 37 °C to allow the compounds to reach their equilibrium solubility. Subsequently, 1 mL fractions were centrifuged in a microcentrifuge at 37 °C and 13,200 rpm for 30 min. The supernatant of each tube was removed, diluted 1:1 with methanol/water 50:50, and quantified by reverse-phase high-performance liquid chromatography (HPLC, see below) to estimate the FP solubilities in the listed dissolution media. Solubility measurements were performed in sextuplicate.

### 2.4. Particle Collection

Collection of single-sized fractions through NGI: To evaluate whether the dissolution method is sensitive to particle size differences, FP-MDI was actuated once into a next-generation impactor (NGI) through a standard USP induction port (Copley Scientific^®^, Nottingham, U.K.). Airflow (28.3 L/min) was controlled by a high-capacity vacuum pump (Copley^®^ HCP5, Copley Scientific^®^, Nottingham, U.K.). A 24 mm glass microfiber filter paper (GF/C™) was placed on stage 2, 4, or 6 of the NGI to collect the deposited drug particles. The filter paper was immediately transferred to the donor compartment of the Transwell^®^ system, with the drug-containing side of the filter paper facing upwards.

Collection of ex-throat fractions: The outlet of a medium-sized VCU throat model was connected to a custom-made dose collection unit containing a 24 mm glass microfiber filter paper (Whatman GF/C™). Airflow (60 L/min) was controlled by the Copley^®^ HCP5 high-capacity vacuum pump. A customized silicone mouthpiece adapter was utilized for the DPI device to provide an air-tight connection between the inhalation device and the throat model. To reduce particle bouncing and the re-entrainment of particles into the airflow, the throat model was coated twice with 3 mL of Molykote™ 316 Silicone Release Spray. After each coating step, the throat was left on the bench for 10 min to allow for solvent and propellant evaporation. Investigational FP-DPI formulations (A, B, or C) were delivered through the mouth–throat model at a flow rate of 60 (L/min). The ex-throat fractions were collected onto the Whatman GF/C™ filter papers. The filter papers obtained from the dose collection step were immediately transferred to the Transwell^®^ system. For the majority of the experiments, one capsule of the DPI formulation of interest or one puff of the MDI was deposited. To investigate the so-called “mass effect”, 1–3 capsules of the investigational DPI-formulations C were used.

### 2.5. Visualization of Deposition Patterns

To visualize and compare the deposition patterns of the particles deposited on the filter paper, either through the NGI or the VCU throat approach, empty gelatin capsules were filled with 13.5 mg of alizarin dye powder and actuated at 60 L/min airflow on GF/C™ filters through each of these collection methods. For the NGI apparatus, only stage 4 was evaluated, with a cut-off diameter of 1.66 μm.

### 2.6. Transwell^®^ Dissolution Studies

Dissolution experiments were carried out at 37 °C and 100% humidity inside a cell incubator. Six-well Transwell^®^ plates with 24 mm polycarbonate membrane inserts and 0.4 µm membrane pore size (Figure 1) were selected, with only one of the middle wells (receptor compartment) utilized per plate. To account for any negligible influence of differences in temperature, stirring speed, and humidity on the study results due to the position of a given plate inside the incubator, experiments were performed in sextuplicate, with replicates positioned at different locations within the incubator.

While, in most of the experiments, the filter paper surface containing the drug was placed downward onto the semi-permeable Transwell^®^ membrane, a set of dissolution experiments with DPI formulation B, collected through the anatomical throat, compared the effects of filter positioning (“face up or downwards”). The “face-up” position was also used in early experiments for Flovent^®^ HFA MDI in experiments evaluating the effects of particle size on the dissolution rate, using material collected at defined stages of the NGI (see above).

A total of 1.5 mL of the preheated dissolution medium (0.5% (*w*/*v*) Tween^®^ 80 in distilled water, or 0.5% (*w*/*v*) SDS in distilled water) was added to one of the middle wells of the Transwell^®^ plate. This receptor compartment was agitated by a stirring bar (length: 7 mm, diameter: 0.78 mm) at 200 rpm. To compare the discriminatory ability of 0.5% (*w*/*v*) Tween^®^ 80 with that of 0.5% (*w*/*v*) SDS, FP-MDI particles collected on stages 2, 4, and 6 of the NGI were studied in both media. Following the placement of the filter paper containing a Transwell^®^ insert (donor compartment), dissolution experiments were initiated by adding 580 µL of the dissolution medium to the donor compartment.

The six individual experiments evaluated on a given day were started one minute apart. This provided sufficient time for subsequent samplings and medium replenishment. Samples of 500 µL were taken from the receptor compartment at pre-specified timepoints of 10, 20, 30, 45, 60, 90, 120, 180, 240, 360, 480, 720, and 1440 min, and substituted with 500 µL of fresh pre-heated dissolution medium. After removing the last sample, the donor compartment was washed twice with 4 mL of methanol, and subsequently diluted with distilled water to obtain a 10 mL “donor wash” sample.

All samples were quantified by reversed-phase high-performance liquid chromatography (HPLC, see below). Sample concentrations were used to calculate the amount of drug that was dissolved and transferred into the receptor compartment at a given sampling timepoint and over the 24 h of the experiment. The cumulative amount of drug that was transferred into the receptor, and the undissolved amount left in the donor compartment, were taken into account when performing the calculations in Excel. Mean dissolution times (MDT) were derived in R (v 3.5.2) [24] by fitting the dissolution profiles to the Weibull function, which was adapted to characterize the dissolution profiles [25,26]:

(1) $$Y_{t}=100\times (1-\exp\left(-{\left(\frac{t}{MDT}\right)}^{b}\right)$$

where *Y_t_* is the percentage of drug dissolved and transferred into the receptor compartment at timepoint *t*; *t* is the sampling timepoint; *MDT* is the in vitro mean dissolution time; *b* is the shape parameter.

To derive the diffusion rate of FP across the insert’s membrane, experiments were performed without the filter paper by adding 580 µL of the saturated FP solution in 0.5% (*w*/*v*) Tween^®^ 80 into the inserts as the donor compartment. Sampling was performed from the receptor compartment at the pre-specified timepoints of 10, 20, 30, 45, 60, 90, 120, 180, and 240 min. All other experimental conditions were identical to those of dissolution experiments.

### 2.7. High-Performance Liquid Chromatography (HPLC) Analysis

Samples obtained from the diffusion and dissolution experiments were diluted 3:7 with methanol:water (50:50), while the donor wash mixture was diluted 1:1 with methanol:water (50:50). FP concentrations were quantified by a reverse-phase HPLC method using a Waters^®^ HPLC system (Waters Co, Milford, MA, USA) with a 717 plus Autosampler (Waters Co., Milford, MA, USA), UV detection at 254 nm (Waters 2487 Dual λ Absorbance Detector (UV/Vis)), a Waters C18 Symmetry column, (100Å, 3.5 µm, 4.6 mm × 30 mm) and an injection volume of 100 µL. A mixture of acetonitrile:water (70:30), at a 1 mL/min flow rate, was selected as the mobile phase. The method, providing a limit of quantification of 0.2 µg/mL, was validated with intra-day and inter-day variabilities of less than 15% and an R^2^ larger than 0.998 for all the calibration curves.

### 2.8. In-Silico Model Development

In silico simulations describing the dissolution, subsequent diffusion, and sampling steps during the Transwell^®^ dissolution experiments were performed in R (v 3.5.2). Differential equations were solved with ‘deSolve’ and ‘minpack.lm’ packages in R, with dt = 0.01 for dissolution experiments and dt = 0.005 if only the diffusion processes of solutions were modeled. The dissolution of the drug particles in the donor compartment was described by the Nernst Brunner equation, Equation (2), while the diffusion of the dissolved drug into the receptor compartment was expressed by Fick’s first law of diffusion Equation (9).

The particle size distribution (PSD) of the sample was integrated into the model as a summation of mono-disperse particles using NGI stage deposition information (amount of drug on stages i = 1 to 7, with each stage using a calibrated cut-off diameter) for DPI formulation C [24]. The amounts associated with each given stage were adjusted for a given dissolution experiment based on the difference between the cumulative amount in NGI stages 1 through 7 and the total drug used in the dissolution experiment. The dissolution of a drug associated with a given stage (bin) was modeled independently, by the Nernst Brunner equation, Equation (1), with all bins contributing to the sum of dissolved drug in the donor compartment, as described by May et al. [18]. The amount of drug deposited at a given NGI stage was considered to represent a bin with a defined calibrated cut-off diameter. Accordingly, Equation (2) was used to describe the dissolution of particles associated with a given bin in the donor compartment:

(2) $$\frac{dX\left(i\right)}{dt}=\frac{-D\times SA_{i}\left(t\right)\times \left(Cs-\frac{X_{d}}{V_{d}}\right)}{r_{i}\left(t\right)}$$

where *X_i_* is the total amount of undissolved drug (g) of a given bin; *D* is the diffusion coefficient (cm^2^/h; see also Equation (8)); *SA_i_* is the surface area of each particle size bin (cm^2^); *C_s_* is the saturation solubility of the compound in dissolution medium (g/mL); *X_d_* is the amount of dissolved drug at time t (g) in the donor compartment; *V_d_* stands for the donor volume (mL); *r_i_* is the diffusion layer thickness, which is equal to the radius of the particles of a given bin (cm) [27].

As the geometric particle size, not the aerodynamic particle size, is relevant for the dissolution process, the aerodynamic particle diameter, associated with a given stage of NGI, was converted into the geometric particle diameter by assuming a spherical shape for the particles, as described by May et al. [18].

The changing surface area and radius of the particles at each timepoint were calculated using Equations (3)–(7), which were used as the input for Equation (2) to describe the dissolution process in the donor compartment.

(3) $$d_{geo}=d_{aero}\sqrt{\frac{k}{\rho }}$$

(4) $$r=\frac{d_{geo}}{2}$$

(5) $$r_{i}\left(t\right)={\left(\frac{3X_{0i}\left(t\right)}{4\pi \rho N_{i}}\right)}^{\frac{1}{3}}$$

(6) $$N_{i}=X_{0i}\left(t=0\right){\left(\frac{4\pi r{\left(t=0\right)}^{3}\rho }{3}\right)}^{-1}$$

(7) $$SA_{i}\left(t\right)=N_{i}4\pi r_{i}{\left(t\right)}^{2}$$

where *d_geo_* is the geometric particle diameter (cm); *d_aero_* is the aerodynamic particle diameter (cm); *k* is the shape factor; ρ is the density of particles (g/cm^3^); *i* is the NGI bin number; *r* is the radius of the particles at a given timepoint, associated with a given NGI bin (cm); *X*_0_ is the undissolved amount of drug of each NGI particle size fraction (bin) in the donor compartment at a given timepoint (g); *N* is the number of particles of each NGI bin or particle size fraction; *SA* stands for the surface area of particles at a given timepoint of each NGI bin or particle size fraction (cm^2^). The radii of the particles and their surface areas were updated at each timepoint by calculating the change in the dissolved amount and, consequently, the reduction in the radius and surface area of the particles at each time, t. the shape of the particles was assumed to be spherical (k = 1) [28]. The density of FP particles was found to be 1.37 g/cm^3^ based on the ChemBK/CAS database [29].

Based on the Hayduk–Laudie equation, Equation (8), with *η_water_* as the viscosity of water at 37 °C and *V_M_* as the molecular volume of FP, obtained according to the approach described by Zhao et al. [30]), the diffusion coefficient *D* of the non-ionized FP was set as 0.0003714 cm/h.

(8) $$D=\frac{13.26\times 10^{-5}}{\;\eta_{water}^{1.4}\times V_{M}^{0.589}}$$

In the Nernst–Brunner model, the entire surface area of each particle is assumed to be surrounded by the dissolution medium, and particles are assumed to be freely suspended in the dissolution medium. As further outlined in the discussion, the Transwell^®^ system differs from this situation, and some parameters in the Nernst–Brunner equation are likely to be affected. Thus, a correction factor (F) was added to the Nernst–Brunner model (not shown in Equation (2)) and determined by fitting the entire simulation model (dissolution and diffusion) to the dissolution data of Formulation C, with F as the only unknown parameter.

The diffusion of the drug across the Transwell^®^ membrane was described by Fick’s first law of diffusion. This step was modeled by Equations (9)–(12), when a solution was introduced into the system.

At each sampling timepoint, the amount of drug removed from the receptor compartment through sampling was accounted for by Equation (11). The cumulative amount of the drug removed through samples at each sampling timepoint was calculated using Equation (12). The only unknown parameter, permeability coefficient (P), was estimated by fitting the observed mean diffusion profile of a solution using Equations (9)–(12).

(9) $$D=\frac{13.26\times 10^{-5}}{\;\eta_{water}^{1.4}\times V_{M}^{0.589}}$$

(10) $$\frac{dXd}{dt}=-P\times SM\times \left(\frac{X_{d}}{V_{d}}-\frac{Y}{Vr}\right)$$

(11) $$\frac{dY}{dt}=P\times SM\times \left(\frac{X_{d}}{V_{d}}-\frac{Y}{Vr}\right)$$

(12) $$Y_{t+∆t}=Y_{t}-\left(\frac{Y_{t}}{Vr}\times Vs\right)$$

(13) $$S=X_{initial}-\left(X_{d}+Y\right)$$

where *X_d_* is the amount of dissolved drug in the donor compartment at a given timepoint (µg); *Y* is the amount of dissolved drug in the receptor compartment at a given timepoint (µg); *P* is the permeability coefficient (cm/min); *SM* is the surface area of the Transwell^®^ membrane (4.52 cm^2^); *V_d_* is the donor volume (mL); *V_r_* is the receptor volume (mL); *V_s_* is the sampling volume; ∆*t* represents the infinitesimal time interval post-sampling event; *S* is the cumulative amount of drug removed at each sampling timepoint (µg); *X_initial_* denotes the total amount of drug in the system.

Equation (9) was substituted by Equation (13) when solid particles were introduced into the donor compartment. Here, the change in the amount of dissolved drug in the donor compartment at timepoint t (*dX_d_*/*dt*) was expressed as the difference between the rate of dissolution and diffusion across the Transwell^®^ membrane:

(14) $$\frac{dX_{d}}{dt}=\frac{\;D\times SA_{i}t\times \left(Cs-\frac{X_{d}}{V_{d}}\right)}{r_{i}t}-P\times SM\times \left(\frac{X_{d}}{V_{d}}-\frac{Y}{V_{r}}\right)$$

The above model was implemented in a user-friendly interface using R shiny to facilitate the efficient optimization of Transwell^®^ dissolution experiments (http://coplin8.cop.ufl.edu:3838/TranswellDissolution/; accessed on 19 July 2021).

### 2.9. Application of the Model

All simulations or model fits were carried out using the ‘deSolve’ package in R software (v 3.5.2), based on the model described above. All simulations were based on the PSD of the investigational FP-DPI, formulation C.

To fit the model to the experimental data, all input parameters (amounts of FP deposited on the filter paper, solubility of FP in the dissolution medium, FP’s permeability across the Transwell^®^ membrane, PSD information of DPI formulation C (see: In Silico Model Development)) were obtained experimentally. The only parameter left to be determined was correction factor F.

During simulations, the target parameter (e.g., solubility, delivered drug amount) was allowed to vary within a specific range, while all the other model parameters were kept constant. The sampling timepoints were those used in the bench experiments, unless stated otherwise. The mean dissolution time (MDT) for each dissolution profile was determined by fitting the Weibull function to the simulated data using the ‘nls’ function in R software (v 3.5.2), as described in Equation (1).

The following simulations were performed:

(1) To determine the solubility below which the transfer profiles obtained for particles would differ from those of a solution, “transfer” profiles (dissolution + diffusion) were simulated for particles differing in solubility (40 µg/mL to 500 µg/mL). These profiles (test) were compared with the diffusion rate profile of dissolved drug (reference), while keeping all other model parameters constant. Difference factor (*f*1) and similarity factor (*f*2) were calculated (Equations (14) and (15) [31]), and two dissolution profiles were only considered to be significantly different from each other if both *f*1 > 15 and *f*2 < 50.
  (15) $$f1=\left[\;\frac{\sum_{t=1}^{n}\left|R_{t}-T_{t}\right|}{\sum_{t=1}^{n}R_{t}}\right]\times 100$$
  (16) $$f2=50\times log\{{\left[\;1+\frac{1}{n}\overset{n}{\underset{t=1}{\sum }}{\left(R_{t}-T_{t}\right)}^{2}\right]}^{-0.5}\times 100\}$$
  *R_t_* and *T_t_* are the mean cumulative percentage of drug dissolved at each timepoint for the reference and test profiles, respectively.
(2) To evaluate the effect of donor volume on the dissolution/transfer rate, the dissolution profile of formulation C was predicted for six different donor volumes (0.1, 0.2, 0.5, 1, 2, and 3 mL) and their corresponding receptor volumes (see Equation (16)), while keeping all the other model parameters constant. The concentration-time-course of dissolved drug particles in the donor and receptor compartments, and the MDT at different donor volume values, were computed;
(3) Similarly, to assess the effect of sampling volume on the dissolution/transfer rate, the dissolution profile of FP DPI particles was determined for six sampling volumes (0.1, 0.2, 0.5, 1, 1.5, and 2 mL), while keeping sampling frequency and all other model parameters constant. The time-course of drug concentration in the donor and receptor compartments, and the MDT at different sampling volume values, were computed;
(4) The ability of the Transwell^®^ setup to differentiate between samples of different particle sizes, and the dependence on the choice of the dissolution medium, was assessed in a range of solubilities (1–20 µg/mL). Dissolution profiles were simulations based on DPI formulation C, and compared with the profiles obtained for hypothetical formulations differing in PSDs from formulation C by a factor of 0.5 and 2. The effect of solubility was assessed using a V_d_ of 0.58 mL, V_r_ of 1.5 mL, and a V_s_ of 0.5 mL);
(5) Lastly, the relationship between the initial amount of FP delivered to the Transwell^®^ setup and the estimated MDT of the drug was studied to evaluate the “mass effect” in the system. Simulations were performed for the following three scenarios: Scenario 1: small donor volume and small sampling volume (V_d_ = 0.58 mL, V_r_ = 1.5 mL, V_s_ = 0.5 mL); Scenario 2: large donor volume and large sampling volume (V_d_ = 3.0 mL, V_r_ = 3.31 mL, Vs = 1.0 mL); Scenario 3: large donor volume and small sampling volume (V_d_ = 3.0 mL, V_r_ = 3.31 mL, _Vs_ = 0.5 mL). For each scenario, dissolution profiles were simulated at six initial dose values of 10, 20, 30, 40, 50, and 60 µg.

## 3. Results

### 3.1. Dimensions of the Transwell^®^ System

The 3D metrology assessment, in conjunction with image reconstruction, provided volume and height estimates of defined spaces within the Transwell^®^ system (Table 1).

Based on this information, Equation (16) was derived, allowing for calculation of the volume in the receptor compartment that is necessary to achieve hydrodynamic balance for a given donor compartment volume.

(17) $$V_{r}=\left(\left(\frac{V_{d}+V_{f}}{0.47}\right)\times 0.35\right)+0{.86+W}_{v}$$

where V_r_ is the volume of the dissolution medium in the receptor compartment; V_d_ is the volume of the dissolution medium in the donor compartment; V_f_ is the filter paper volume (0.118 mL); W_v_ accounts for the volume of dissolution medium required to wet the polycarbonate membrane (~100 µL).

### 3.2. Particle Deposition Pattern during Dose Collection

**Figure 2:**
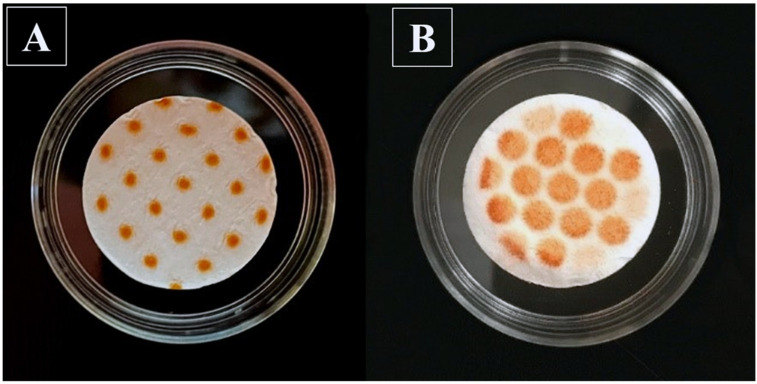
Deposition pattern of alizarin dye powder; (**A**) NGI (stage 4); (**B**) VCU mouth-throat.

The deposition patterns of alizarin dye, when collected through the VCU mouth-throat model or stage 4 of the NGI impactor (Figure 2), indicated that the ex-throat particles deposit more evenly on a larger filter surface. In contrast, with the NGI approach, particles accumulate on filter paper on a smaller surface, close to the air jet outlets, indicating potential pile formation.

### 3.3. Filter Paper Positioning

**Figure 3:**
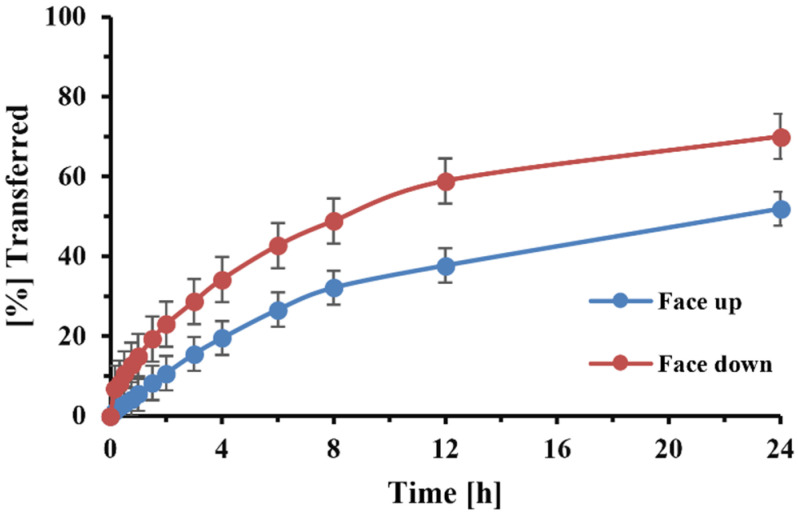
Effect of filter paper position (“face up” or “face down”) on the transfer rate of FP after deposition of 1 capsule of DPI formulation B using the anatomical VCU mouth–throat model for sample collection. The error bars indicate the standard deviation of three replicates.

Figure 3 illustrates the observed difference between the transfer profiles of FP-DPI particles (formulation B) when samples were presented “face up” or “face down”. The MDTs for the face-up and face-down positioning were 33.7 (±3.1) and 15.4 (±0.8) h, respectively.

### 3.4. Establishing the In Silico Model

**Figure 4:**
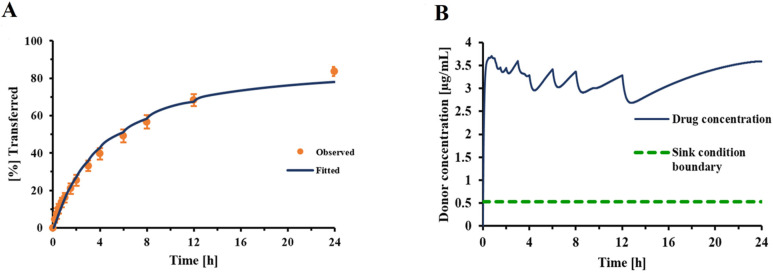
(**A**): Experimentally observed dissolution profile of FP-DPI formulation C (●, error bars indicate the standard deviation of six replicates) and model fitted profile (navy blue line); (**B**) Model predicted dissolved FP concentration-time profile in the donor compartment; inputs: V_d_ = 0.58 mL, V_r_ = 1.5 mL, V_s_ = 0.5, solubility of 5.3 µg/mL, drug amount: 23 ± 2.4 µg, PSD was based on published NGI cascade impactor data [24].

By fitting the mean data obtained from diffusion experiments for FP solution (n = 4) to Fick’s first law of diffusion, the permeability coefficient associated with the transfer of dissolved FP across the Transwell^®^ membrane was estimated to be 0.289 cm/h. Using this information, a correction factor (F) of 0.0244 (±8.95% RSE) was determined for the dissolution profile of the investigational FP-DPI formulation C, resulting in a good agreement between the model-derived and observed dissolution profiles (Figure 4A). The resulting concentration-time course of dissolved FP in the donor compartment is illustrated in Figure 4B.

### 3.5. Varying Donor/Receptor Compartment and Sampling Volumes

**Figure 5:**
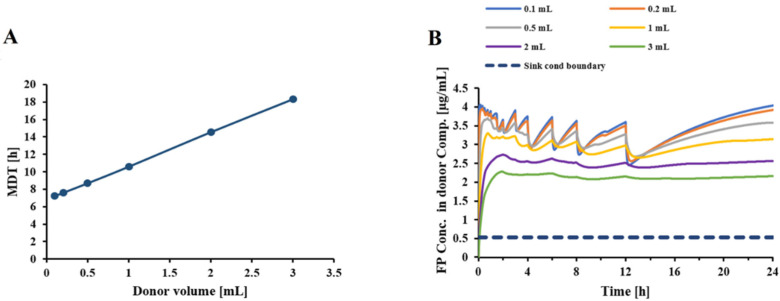
(**A**) Impact of donor/receptor volumes on the MDT of FP (formulation C) in the Transwell^®^ setup; (**B**) Related dissolved FP concentration profiles in donor compartment (each color represents a donor volume, which was paired with the corresponding receptor volume based on Equation (16)); Inputs: Drug amount: 20 µg, V_s_: 0.5 mL, solubility of 5.3 µg/mL, Particle size information of formulation C was based on published NGI cascade impactor data [24].

**Figure 6:**
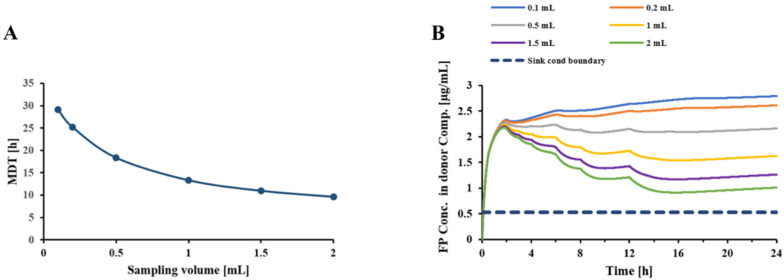
(**A**) Impact of sampling volume on MDT of FP; (**B**) Related dissolved FP concentration profiles in donor compartment; Inputs: Drug amount: 20 µg, V_d_ = 3 mL, V_r_ = 3.31 mL, PSD was based on published NGI cascade impactor data for formulation C [23], solubility = 5.3 µg/mL.

By applying the developed model for FP-DPI formulation C, dissolution profiles were simulated for a range of donor/receptor volumes. The resulting MDTs are shown in Figure 5A, while the corresponding dissolved FP concentrations in the donor compartment are depicted in Figure 5B. Figure 6A,B displays how the sampling volume affects the MDT and FP donor concentrations, respectively.

### 3.6. “Mass Dependency” of the Dissolution Process in Transwell^®^ Setup

**Figure 7:**
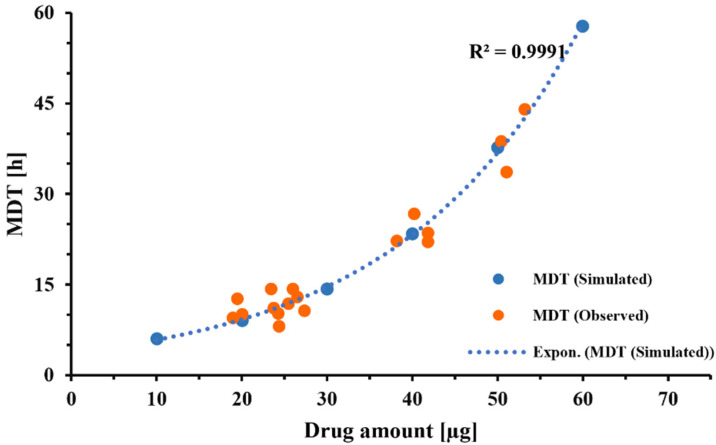
MDT vs. amount of FP introduced to the Transwell^®^ dissolution setup. Orange symbols: experimentally observed MDTs; Blue symbols and dotted line: Model predicted estimates; Inputs: V_d_ = 0.58 mL, V_r_ = 1.5 mL, V_s_ = 0.5 mL. Drug amount refers to the amount of drug present in the donor compartment at the start of the experiment.

**Figure 8:**
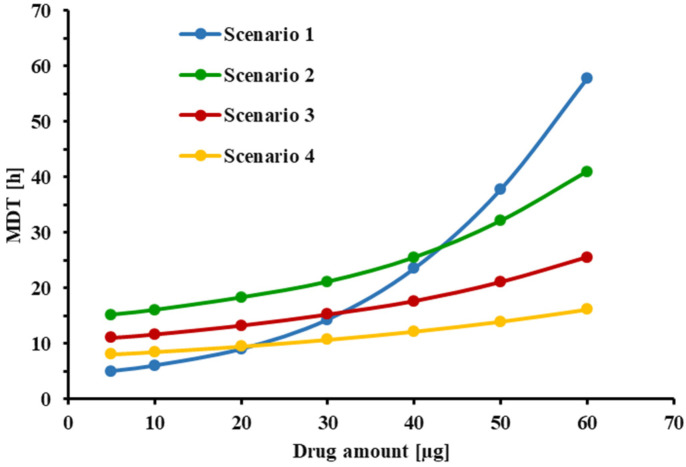
Effect of drug amount on MDT of FP in four different scenarios: Scenario 1: V_d_ = 0.58 mL, V_r_ = 1.50 mL, and V_s_ = 0.5 mL; Scenario 2: V_d_ = 3.00 mL, V_r_ = 3.31 mL, and V_s_ = 0.5 mL; Scenario 3: V_d_ = 3.00 mL, V_r_ = 3.31 mL, and V_s_ = 1.0 mL. Scenario 4: V_d_ = 3.00 mL, V_r_ = 3.31 mL, and V_s_ = 2 mL. Solubility in all scenarios: 5.3 µg/mL. Drug amount refers to the amount of drug present at the start of the experiment in the donor compartment.

Figure 7 illustrates the experimentally determined and model-predicted MDTs of formulation C when 1–3 capsules were collected through the VCU mouth-throat model. Figure 8 displays how experimental conditions affect the extent of the mass effect by evaluating scenarios that differ in terms of donor/receptor and sampling volumes. Using the MDT obtained after depositing 10 and 30 µg drug volumes onto the filter paper as comparators, MDT_30µg_/MDT_10µg_ ratios for scenarios 1–4 were estimated to be 2.38, 1.35, 1.31, and 1.25, respectively. If more frequent sampling (hourly intervals starting at 2h) was employed, this ratio would be further decreased for scenarios 1 and 4, to 1.8 and 1.2, respectively.

### 3.7. Identification of a Suitable Dissolution Medium

Table 2 lists the experimentally determined solubilities of FP in a range of SDS, Tween^®^ 80, and diluted Survanta^®^ dissolution media.

**Figure 9:**
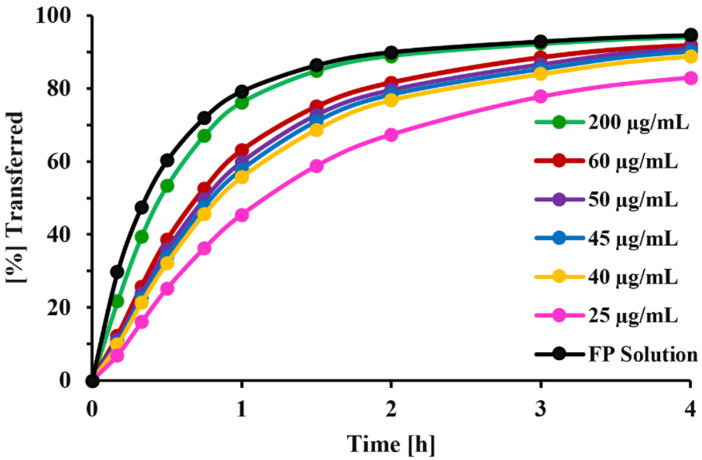
Effect of solubility on dissolution profile of FP-DPI (formulation C); comparison with diffusion profile of FP solution; Inputs: Drug amount: 23 µg, V_d_ = 0.58 mL, V_r_ = 1.50 mL, V_s_ = 0.5 mL.

Figure 9 demonstrates the effect of solubility on dissolution profiles of FP. Based on f1 and f2 tests, the dissolution profiles of FP solid particles (Formulation C) will be significantly different from that of an FP solution for solubilities below 45 µg/mL, with the mean transfer time being about twice that of the solution (Table 3).

**Figure 10:**
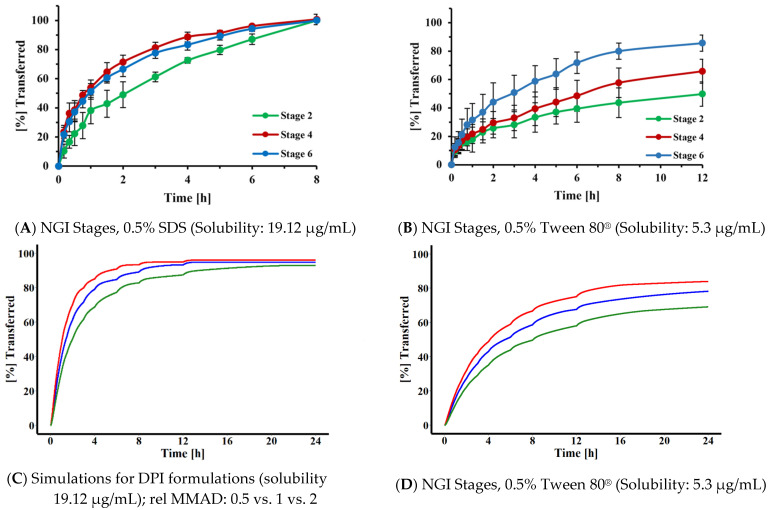
Dissolution profiles of Flovent^®^ HFA MDI particles collected on NGI stages 2, 4, and 6 in (**A**) 0.5% (*w*/*v*) SDS and (B) 0.5% (*w*/*v*) Tween 80^®^ in water. Filter was positioned “face up”. (**C**) Simulations for polydisperse DPI formulations assuming a solubility of 19.12 µg/mL; (**D**) Simulations for polydisperse DPI formulations assuming a solubility of 5.3 µg/mL. For both simulations, relative MMADs of 0.5 (red), 1 (blue) or 2 (green) were selected; Drug amount: 23 µg; V_d_ = 0.58 mL, V_r_ = 1.50 mL, V_s_ = 0.5 mL.

The impact of the dissolution medium on the ability of the method to distinguish samples differing in particle size was assessed experimentally by comparing the dissolution profiles of FP particles (Flovent^®^ HFA MDI) that were collected on NGI stages 2, 4, and 6 (representing particles with aerodynamic cut-off diameters of 6.4, 2.3, and 0.83 μm, respectively). As indicated in Figure 10A,B, the medium providing the lower solubility for FP (0.5% Tween^®^ 80, 5.3 µg/mL) was more able to differentiate between particles of different sizes than the medium exhibiting higher solubility for FP (0.5% SDS, 19.12 µg/mL). These experimental results were confirmed for polydisperse samples through simulations (Figure 10C,D). Dissolution profiles were simulated for several dissolution media, providing different drug solubilities (19.12, 15, 10, 5.3 and 1.23 µg/mL). Simulations suggested that, for three samples with relative particle sizes of 0.5, 1 and 2, the best resolution was achieved for the dissolution medium providing a solubility of about 5 µg/mL (see Figure 10C,D for scenarios evaluating solubilities of 5.3 and 19.12 µg/mL), resulting in MDTs being roughly 10 times longer than the mean transfer time of a solution; 7 h vs. 0.6 h).

**Figure 11:**
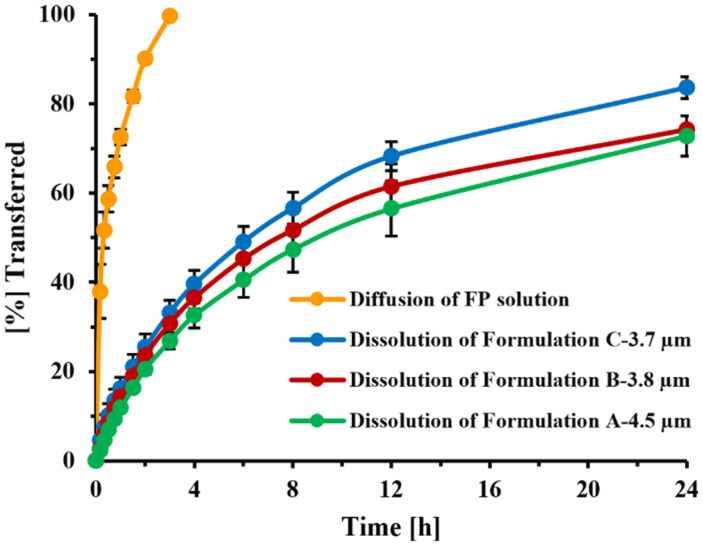
Mean diffusion and dissolution profiles of FP solution and FP-DPI formulations observed in Transwell^®^ setup for formulation A-4.5µm, B-3.8µm C-3.7µm. The error bars indicate the standard deviation of four replicates for diffusion and six replicates for dissolution profiles.

Utilizing the described dissolution setup, and using the lower-solubility Tween 80^®^ (0.5%) as dissolution medium, dissolution profiles of the three investigational FP-DPI formulations with different PSDs [23] were compared with the transfer rate of dissolved FP (Figure 11). The results confirmed the sensitivity of the selected method to detect differences in MMAD. As shown, the diffusion of dissolved FP particles was significantly faster than the dissolution of the solid drug particles, with a mean diffusion time of 33.6 min for FP solution and mean transfer times of 15.4, 13.3, and 10.3 h for formulations A (MMAD: 4.5 µm), B (MMAD: 3.8 µm), and C (MMAD: 3.7 µm), respectively.

## 4. Discussion and Conclusions

In a continuation of previously published work [21,22], we further optimized and evaluated the Transwell^®^ system to assess the dissolution behavior of OIDPs. This included the use of a more relevant aerosol collection method.

While dissolution tests have been performed on OIDPs with “unfractionated” material, applied via airbrush, dry powder insufflators or aerosol generators [6], most stakeholders agree that tests should be performed with a fraction of the delivered dose, resembling the material deposited in the lung of patients as closely as possible [5]. As an example, samples were collected on relevant cascade impactor stages [21,32], or prepared with abbreviated or modified cascade impactors [13,17,18]. It has been suggested that the so-called mass effect, the slow-down in dissolution rate with an increase in the amount of drug used [7,12,13,18,32], might be, at least in part, due to the formation of drug piles of agglomerated material underneath the nozzles of the cascade impactor stages. May et al. used an abbreviated Anderson cascade impactor (ACI) with a spacer to deposit the drug particles more uniformly after impaction [10]. Price et al. modified the NGI cascade impactor to uniformly deposit the whole impactor stage mass (ISM) on a glass fiber filter [17]. This study employed an anatomical mouth–throat model, in conjunction with a custom-made filter holder, to collect relevant samples in vivo. This method was selected because Olsson et al. showed a good agreement between the in-vitro-derived ex-throat dose and the drug amount deposited in the lungs of human subjects [33]. The pattern of collected alizarin powders through the VCU mouth–throat model showed a more uniform dispersion of particles over a larger filter paper surface compared to the NGI.

We decided to coat the throat, to reduce particle bouncing at flow rates of 60 L/min. Future studies might employ typical inhalation profiles instead of constant flow rates [33]. As indicated in Figure 2B, the use of an anatomical throat resulted in a more uniform dispersion of particles over a larger filter paper surface compared to that of the NGI (Figure 2A), with the honeycomb shapes caused by the structure of the metal support holding the filter paper. We decided to use the ex-throat methodology in subsequent experiments, without further modifications. Future studies should also evaluate how the choice of anatomical mouth–throat model (VCU, Alberta, Consortium), size (small, medium, or large), and alternative flow rates (e.g., simulations of actual inhalation profiles) might affect the dissolution profiles. While it was hoped that the use of anatomical models for sample collection would eliminate the mass effect, the results given below show that this was not fully successful; the effect was also related to the conditions within the Transwell^®^ system.

Previously, Rohrschneider et al. [21] observed that the use of an unmodified Transwell^®^ system (i.e., 0.4 µm polycarbonate membrane) was unable to distinguish between the transfer rates of Ciclesonide particles and those of a solution if the receptor compartment was not stirred. This resulted in the replacement of the 0.4 μm pore size membrane of the Transwell^®^ system with a more permeable filter paper. This approach involved the engineering of thermo-formed notches to support the filter paper. The reproducible placement of the drug-containing filter paper was a challenge in routine use. In the current study, we evaluated whether keeping the commercial membrane could be a viable alternative if equilibrium between the donor and receptor compartments could be accelerated by stirring the receptor compartment. We continued using a 0.4 µm pore size to ensure that undissolved micronized particles were unlikely to enter the receptor compartment. The polycarbonate membrane was selected rather than the polyester membrane because of the higher pore density (i.e., 25 times higher) [16].

The employed reverse-phase HPLC method provided a quantification limit of 0.2 µg/mL. The need for higher sensitivities, e.g., for products administered at much lower doses or for substances that cross the Transwell^®^ membrane at significantly lower rates, might require more sensitive analytical methods, such as HPLC-MS/MS.

In the current Transwell^®^ setup, diffusion across the 0.4 µm PC membrane was facilitated by stirring of the receptor compartment. Under these conditions (7 mm stirring bar, 200 rpm stirring speed), the transfer of dissolved FP (time to transfer 60% of dissolved drug: 30 min, Figure 11) was slightly slower than that observed by Rohrschneider et al. in the modified Transwell^®^ system (use of filter paper instead of Transwell^®^ membrane, no stirring, time to transfer 60% of dissolved drug: 15 min [21]), but much faster than what was observed under the original conditions (0.4 µm PC membrane, filter paper face-up, no stirring, time to transfer 60% of the drug: 2.5 h, [21]). Nevertheless, the method was able to clearly distinguish between the transfer rates of solution and particles (Figure 11). The agitation speed of the custom-made stirring bar was selected as high enough to ensure sufficient mixing, but low enough to prevent foam formation and uncontrollable movements of the stirring bar. Studies evaluating how differences in agitation speed would affect dissolution profiles were not performed, especially as the effects of stirring speed on the stagnant layer of particles immobilized within the spatially restricted space between the filter paper and Transwell^®^ membrane were assumed to be less pronounced than effects on freely suspended particles within a standard USP vessel.

The use of the 0.4 µm membrane further permitted the face down placement of the filter paper without undissolved particles entering the receptor compartment. We also accelerated the dissolution process and shortened the time that was necessary for the experiment (Figure 3). The downward filter position further reduced the variability, as it eliminated the solute’s diffusion across the filter paper. Sakagami et al. [13] positioned the filter paper in a similar way.

To be more flexible with respect to the choice of donor and receptor volumes, detailed information on the dimensions of the Transwell^®^ system was obtained, which allowed for a relationship to be derived that could match the volume of the dissolution medium in the donor and receptor compartments. This allowed for the easy adjustment of the experimental conditions, while ensuring equal fluid levels were maintained in both compartments and preventing fluid transfer due to differences in hydrostatic pressure. The derived relationship, shown in Equation (16), was used to adjust dissolution medium volumes during the subsequent in silico simulations.

To more efficiently assess the effect of experimental conditions (solubility of a compound in a dissolution medium, changes in the volume of donor and receptor compartment, and even the sampling volume) on assay performance, it was of interest to predict the outcome of dissolution experiments in silico. Based on the Nernst–Brunner equation and in accordance with May’s simulation approach [18] to assessing the dissolution of OIDPs with the USP apparatus, the model used a series of differential equations to describe the dissolution of particles of a known aerodynamic particle size distribution in the donor compartment.

In contrast with May’s model, this further integrates dissolution and diffusion steps. Our model further differs from May’s and a recently published model developed by Frenning et al. regarding membrane-based dissolution tests [34], as it considers the sampling procedures within the model. Using the parameters employed by May et al. to describe the dissolution process (diffusion coefficient in the stagnant layer, thickness of the stagnant layer), we were unable to predict the experimental dissolution profiles of DPI formulation C (data not shown). This was not surprising as, within the Transwell^®^ set up, particles are not freely suspended within the dissolution vessel, but embedded in a restricted three-dimensional structure formed by the irregular filter paper surface, with particles further confined by the presence of the sandwich formed between the filter paper and Transwell^®^ membrane. It is, therefore, likely that the thickness of the stagnant diffusion layer, previously shown to depend on stirring rate, and other spatial factors modulating the effective diffusion across this layer, will be different from the one used by May and co-workers [18]. The correction factor also adjusted for differences between the actual water-accessible surface area and that obtained from cascade impactor studies. Differences between these two estimates are due to the fact that, for lactose-containing DPI formulations, the aerodynamic particle size distribution (APSD) is driven, in part, by drug particle/lactose agglomerates, while the calculation of surface area from APSD studies assumes that “pure” drug particles are deposited. In addition, the water-accessible surface area will be affected by the formulation-dependent composition of the agglomerates (e.g., ratio and spatial arrangement of lactose fines and drug particles within an agglomerate). In the proposed dissolution setup, a further reduction in water-accessible surface area might occur via blockage of the drug particles’ surface through direct contact with the filter paper or Transwell^®^ membrane.

Incorporating this correction factor into the Nernst–Brunner equation resulted in a good fit between experimentally observed and fitted dissolution profiles (Figure 4), with all other parameters being derived experimentally or obtained from relevant physicochemical relationships (Hayduk–Laudie equation). Using this correction factor, derived from the experiment depicted in Figure 4, simulations correctly predicted the dissolution behavior of DPI formulation C, over a wide range of drug amounts (Figure 7). This indicated that the correction factor determined from one set of experiments (Figure 4) was relatively constant across experiments as long as the particle-surface-area-related differences were fully reflected in the information the model received from APSD data. Considering this, the correction factor should be less dependent on formulation attributes than the z-factor [35]. However, within our experimental setup, the correction factor was also adjusting for differences between the “true” accessible surface area of the drug particles and the surface area obtained from APSD measurements (see above). Additional studies need to be performed to further quantify the effects of formulation and experimental conditions (e.g., the nature of the filter paper) on correction factor F. The agreement between the experimentally observed and predicted mass effect (Figure 7) supported the validity of the model developed in silico for this specific formulation and justified the model’s use when evaluating changes in experimental conditions.

Rohrschneider et al. employed 0.5% SDS as the dissolution medium during our original assessment of the Transwell^®^ system, without further optimization, as this dissolution medium was able to clearly differentiate between the transfer rates of a model corticosteroid when assessed as a solution or as solid particles. In the present study, we were interested in developing a strategy for selecting a suitable dissolution medium for compounds differing in hydrophilicity. Based on the simulations shown in Figure 9, the MDT of the particles had to be at least three times longer than the transfer time observed for the corresponding solution, in order to differentiate between the solution and a particular FP. This translated into a solubility of 45 µg/mL. Assuming that the target permeability compounds with similar structure were close to the permeability determined in this study for FP (P = 0.289 cm/h), similar relationships would be applicable for most slowly dissolving inhaled corticosteroids. Interestingly, Sakagami et al. identified a similar media strength when empirically adjusting the solubility of the dissolution media for lipophilic corticosteroids such as FP, in their Transwell^®^ system [13]. The projected dissolution medium, however, represents the upper limit. Simulations and experimental data (Figure 10) suggested that the dissolution media with reduced solubility (in the range of 5 µg/mL rather than 20 or 45 µg/mL) would provide higher resolution when identifying differences in particle size of OIDPs. With this reduced solubility, the dissolution profiles of particles deposited on stages 2, 4, and 6 were more distinguishable (Figure 10) than those obtained with the higher-solubility medium. Our results agree with those of Liu et al. [36], who also reported the masking of particle size differences when the dissolution medium provided higher solubilities The need for lower-solubility conditions was also indicated within simulations for polydisperse systems (Figure 10C,D) and the three experimental DPI formulations [23] for which the dissolution behavior differed, although MMAD estimates differed only slightly (Figure 11).

Hence, a dissolution medium providing a solubility of around 5 µg/mL for the drug of interest, resulting in MDTs about 10 times longer than that of a solution, is likely to provide an acceptable resolution for detecting differences in particle size. The use of solubility data such as those shown in Table 2, and those reported for a broader range of compounds provided by Bhagwat et al. [22], might facilitate the identification of a suitable dissolution medium that yields the targeted solubility for a specific compound. A wide range of dissolution media, differing in the complexity and nature of the surfactant and salt composition, has been evaluated [5,22], and our simulations seem to suggest that the performance in the Transwell^®^ system was mainly driven by the overall solubility of the drug in the medium. Although DPPC and Survanta^®^, both of which contain phospholipids, might be seen as more biorelevant, Tween^®^ 80 is less expansive, readily available, and MDTs obtained with Tween^®^ 80 correlated well with the MATs in vivo [22]. It should be noted that the above recommendations for selecting the solubility of dissolution medium (expressed in µg/mL) are only valid for other drugs if their permeabilities across the Transwell^®^ membrane are similar to that of FP. The statement that MDT should be about 10 times longer than the mean transfer times of a solution should still hold true. The above results represent only a starting point for the further optimization of experimental conditions for a specific comparison.

The phenomena of a high drug amount being delivered to a dissolution setup, hindering the in vitro dissolution rate of OIDPs, has been reported for almost all dissolution methods developed for OIDPs [8,13,37,38]. Different approaches have been explored to counter this mass effect. Hypothesizing that this effect is due to pile formation during particle collection, attempts have been made to ensure a more uniform drug [17,18]. Although the modified particle collection method employed in this study was effective in minimizing the pile formation and agglomerations of particles (Figure 2), the results of Figure 7 showed that the mass effect continued to be significant for the small-volume setup (V_d_ = 0.58 mL, V_r_ = 1.50 mL, V_s_ = 0.5 mL), with the experimental results agreeing well with the model predictions (Figure 7). The reason for this mass effect was presumed to be the lack of sink conditions (Figure 4B). The first attempts evaluated whether an increase in donor/receptor or sampling volumes would reduce the mass effect. Figure 5 indicated that an increase in donor/receptor volumes increased the MDT, while slightly reducing the free drug concentrations in the donor compartment. Interestingly, as indicated in Figure 8, increasing the volumes in the Transwell^®^ setup increased the MDT for lower doses, presumably by slowing down the diffusion process because of the reduced concentration gradient between the donor and receptor compartments. Nevertheless, increasing the volumes for higher doses resulted in faster dissolution rates and lower MDTs, by reducing the free drug concentrations and keeping the conditions closer to the sink conditions. Overall, an increase in the donor/receptor volumes reduced the mass effect (compare scenario 1 vs. 2 in Figure 8). This effect was further diminished when the sampling volume was increased. As indicated in Figure 6A, an increase in sampling volume reduces the mean dissolution time through a series of events. First, increasing the sampling volume increases the concentration gradient between the donor and receptor compartments. As a result of the more pronounced concentration gradient, the diffusion rate across the Transwell^®^ membrane is increased. This leads to a reduction in the concentration of dissolved drug in the donor compartment (Figure 6B), thereby approaching sink-conditions around the drug particles. Consequently, a faster dissolution and reduced MDT will be observed. More importantly, the attenuated “lack of sink conditions” will lead to a further reduction in the mass effect (Figure 8), reducing the ratio of MDT_30µg_/MDT_10 µg_ from 2.0 for scenario 1 (V_s_ = 0.5 mL) to 1.35 for scenario 2 (increase in receptor/donor volume only; V_S_ = 0.5 mL),1.31 for scenario 3 (V_s_ = 1.0 mL) and 1.25 for scenario 4 (V_s_ = 2.0 mL). Similar rank orders were obtained when MDT_60µg_/MDT_10 µg_ were compared. Only a slight decrease in mass effect was observed if more frequent sampling was utilized (hourly sampling), resulting in a ratio of 1.20 for scenario 4 (data not shown). Thus, to reduce the mass effect, experiments should be performed with increased donor/receptor and sampling volumes. The mass effect, however, could not be completely eliminated. Considering that, in most of our experiments, the collected drug amount was between 10 and 30 µg, the mass effect under these improved conditions is almost negligible (MDT_30 µg_/MDT_10 µg_ < 1.3).

In conclusion, through a combination of in vitro and in silico work, we have further evaluated the Transwell^®^ system as a potential methodology for assessing the dissolution behavior of OIDPs. This study proposes the use of anatomical throats for dose collection, as this achieved a more uniform distribution of the collected drug particles during sample preparation. The developed simulation module, which is available on the provided net, was validated as an efficient tool to assess and optimize experimental conditions with respect to the choice of dissolution medium and donor/receptor and sampling volumes. Based on these simulations and the experimental work, we propose performing dissolution tests with a medium that will provide solubility within the range of 5–10 µg/mL, resulting in MDTs times about 10 times longer than those of a solution. The work further suggested that the use of larger donor/receptor and sampling volumes could make the system less sensitive to the potential confounding effect of differences in the collected mass. It is, therefore, suggested that for the small Transwell^®^ system a donor volume of 3 mL, a receptor volume of 3.31 mL, and sampling volumes of up to 2 mL be utilized. Simulations assessing the effects of changes in experimental conditions (drug mass at start of the experiment, donor/receptor and sampling volumes) indicated the complexity of the system as a combination of dissolution and membrane diffusion within a volume-limited system under non-sink conditions. Further studies need to comprehensively compare the Transwell^®^ setup with other adapted dissolution methods, such as USP apparatuses, with respect to robustness and sensitivity, to detect differences in formulation attributes for a broader range of lipophilic OIDPs.

## Figures and Tables

**Table 1:** Characteristics of the Transwell^®^ system.

Space	Maximum Volume (mL)	Height (mm)	Volume (mL)/mm Rise ^5^
Donor compartment ^1^	4.52	9.53	0.47
Receptor compartment ^2^	4.18	10.78	-
Receptor_min_^3^	0.86	1.41	-
Receptor compartment minus Receptor_min_^4^	3.32	9.37	0.35

^1^ Donor compartment is measured from the insert’s polycarbonate membrane up to the holes’ level on the insert’s wall. ^2^ This space represents the total available receptor volume from the bottom of Transwell^®^ well to the holes’ level on the insert’s wall. ^3^ This space represents the available space between the insert’s membrane and the bottom of Transwell^®^ well. This is the minimum volume necessary for the receptor compartment to contact the donor compartment. ^4^ This space is measured from the insert’s membrane level in the receptor compartment to the holes’ level on the insert’s wall (Receptor–Receptor_min_). ^5^ The variable “Volume (mL)/mm rise” indicates the volume needed for a 1 mm increase in the height of each compartment.

**Table 2:** Solubility of FP in different surfactant-containing dissolution media at 37 °C.

Surfactant	Concentration (% *w*/*v*)	FP Solubility * (µg/mL)
SDS	0.05	0.73
	0.20	3.63
	0.50	19.12
	1.00	48.59
	5.00	151.81
Tween^®^ 80	0.01	1.23
	0.10	4.21
	0.50	5.30
	1.00	9.18
	5.00	38.97
Survanta^®^	0.2	3.17

* Some of the data were provided graphically in a previous publication [22].

**Table 3:** Difference factor (f1) and similarity factor (f2) when dissolution profiles in different solubility scenarios were compared with that of a solution.

FP Solubility (µg/mL)	MDT (h)	*f*_1_ (Difference Factor)	*f*_2_ (Similarity Factor)	Result of f1/f2 Test Comparison with Solution
Solution	0.59	-	-	-
60	1.11	11.9	46.4	Similar
50	1.22	15.0	42.3	Similar
45	1.30	15.1	41.7	Different
40	1.39	16.4	40.1	Different

## Data Availability

Not applicable.
